# *Senyumiagranitica* (Gesneriaceae) from Johor, Malaysia, the second species of *Senyumia*

**DOI:** 10.3897/phytokeys.117.31560

**Published:** 2019-02-05

**Authors:** Ruth Kiew, Kah-Hoo Lau

**Affiliations:** 1 Forest Research Institute Malaysia, 52109 Kepong, Selangor, Malaysia Forest Research Institute Malaysia Kepong Malaysia

**Keywords:** New species, *Senyumia granitica*, *Senyumia minutiflora*, ecology, conservation

## Abstract

The genus *Senyumia* was previously known from a single species, *S.minutiflora* (Ridl.) Kiew, A.Weber & B.L.Burtt, from a limestone karst, Gunung Senyum, in Pahang, Malaysia. *Senyumiagranitica* Kiew, here described and illustrated, is the second species of the genus. It differs from *S.minutiflora*, not only in its habitat, but also in its shorter leaves, larger, non-resupinate or only partially resupinate flowers and smaller seeds. It is known from a small, fragmented population from a low range of hills. Therefore, under the IUCN Red List Categories & Criteria, it is assessed as Critically Endangered.

## Introduction

While investigating the granite cliffs of Bukit Belading in Johor State, the site at which the new species of cycad, *Cycascantafolia* Jutta, K.L.Chew & Saw, has recently been discovered (Jutta et al. 2010), an unusual species of Gesneriaceae was found that did not match any known species. However, it was clearly closely similar morphologically to *Senyumiaminutiflora* (Ridl.) Kiew, A.Weber & B.L.Burtt in habit, leaf shape, texture and indumentum, white flowers and twisted fruits, but it differs in its larger flowers that are not or are only partially resupinate. *Senyumiaminutiflora* is unique amongst Peninsular Malaysian and indeed amongst Asian gesneriads in its resupinate flowers. Previous to this discovery, *Senyumia* was a monotypic genus, its single species being known from Gunung Senyum, Pahang, an isolated limestone karst hill. With the discovery of this new species, the resupinate flower can no longer be considered a diagnostic character for the genus.

Morphologically, the genus *Senyumia* Kiew, A.Weber & B.L.Burtt most resembles another Malaysian monotypic genus, *Spelaeanthus* Kiew, A.Weber & B.L.Burtt, that is restricted to karst limestone (Kiew et al. 1998). They share thin, pale green leaves with a toothed margin, small, white flowers and short (less than 10 mm long), twisted capsules. Molecular analysis also places *Senyumia* in a well-supported subclade together with *Spelaeanthus* and the Australian species of *Boea* Lam. (Puglisi et al. 2016). *Senyumia* is distinct from *Spelaeanthus* in its larger leaves, corolla that has a very short, straight-sided, slightly dilating tube and the lobes that are in the upper position being strongly reflexed, the anthers are large and project beyond the corolla tube and the capsule that is distinctly twisted. In contrast, *Spelaeanthus* (represented by a single species, *S.chinii* Kiew, A.Weber & B.L.Burtt), has smaller leaves, a corolla with a longer, broadly inflated tube, the upper lobes are not reflexed, the stamens are included within the corolla tube and the capsule is scarcely twisted. On-going molecular work confirms that this new taxon from Johor belongs to *Senyumia* and is distinct from *Senyumiaminutiflora* (C. Puglisi, pers. comm.).

**Figure 1.**
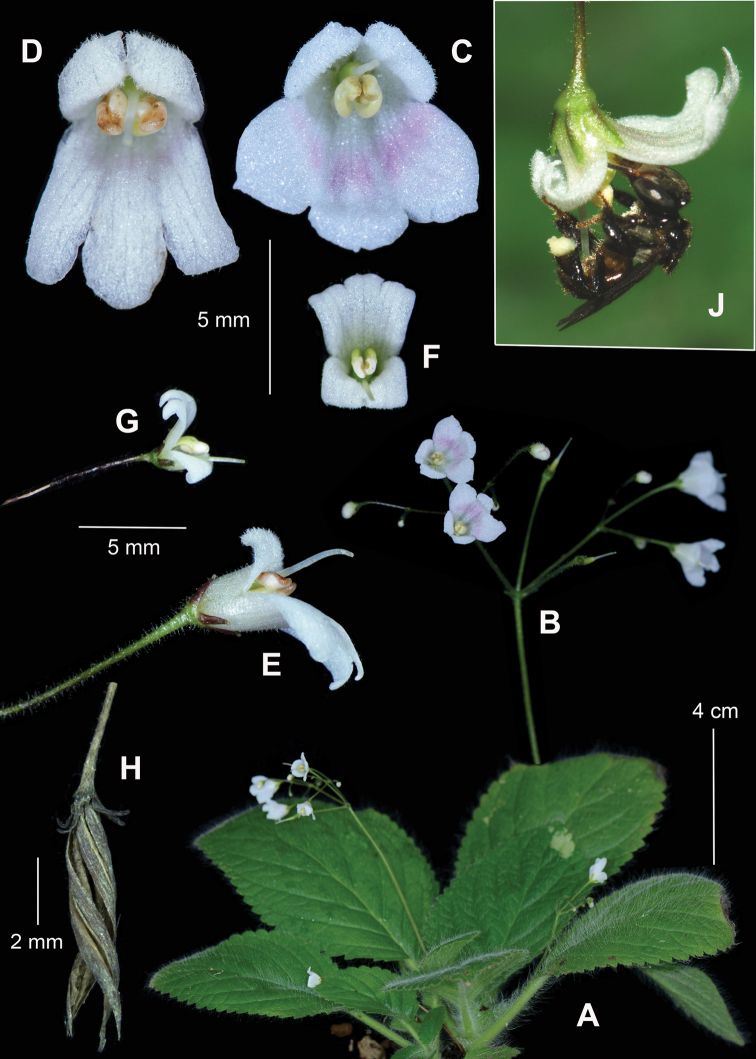
*Senyumiagranitica* Kiew, sp. nov. **A** habit **B** Inflorescence showing partially resupinate flower **C** front view of a flower from Bukit Tukau population **D** front view of a flower from Bukit Reban Kambing population **E** side view of **D**; *Senyumiaminutiflora* (Ridl.) Kiew et al. **F** resupinate flower **G** side view of flower (upside down for comparison with **E**) **H** fruit of *S.granitica***J***Trigonalaeviceps* on *S.minutiflora*, note full pollen baskets. (photographs by PT Ong).

The absence of a nectary and the short corolla tube with large protruding anthers are characters of a pollen flower (Weber 2004). Ong Poh-Teck, Forest Research Institute Malaysia, observed the stingless bee, *Trigonalaeviceps* Smith, visiting flowers of *Senyumiaminutiflora* and collecting pollen (Fig. 1J). Weber (2004) suggested that extremely small flowers and the production of plentiful capsules and seeds in the wild, as is the case in *Senyumiaminutiflora*, may be indicative of autogamy. However, in the Herbarium Nursery at the Forest Research Institute Malaysia, plants grown in an enclosure where insects were excluded, regularly flowered but did not produce fruits (P.T. Ong, pers. comm.) but neither did those flowers visited by the trigona bees that were grown outside the enclosure, suggesting that the trigona bees were pollen thieves rather than pollinators. The role of the resupinate position in *S.minutiflora* is not understood but the three larger basal lobes raised above the flower may serve to make the minute flower more conspicuous to the pollinator (Fig. 1F). The resupinate position does not appear to play a role in access to the flower by the pollinator because the fine pedicel does not support the weight of an insect like trigona. The flower is in a hanging position as the insect removes the pollen (Fig. 1J). However, the great difference in corolla size between the two species suggests that different pollinators are probably involved and the degrees of resupination may be an adaptation to these different pollinators.

## Methods

Materials used for the description of the new species are based on living collections grown in the Herbarium Nursery at the Forest Research Institute Malaysia. However, measurements were made on dried herbarium specimens. Characters of the new species were compared with those of herbarium specimens of similar species in BM, E, K, KEP, KLU, SING and UKMB (herbarium codes from *Index Herbariorum* at http://sweetgum.nybg.org/ih) and living specimens of the new species, *Senyumiaminutiflora* and *Spelaeanthuschinii*. The conservation status of the new species is assessed by the Malaysian regional evaluator, LSL Chua, Forest Research Institute Malaysia, using the standard IUCN Categories and Criteria (IUCN 2012).

## Taxonomy

### Senyumia

Taxon classificationPlantaeLamialesGesneriaceae

Kiew, A. Weber & B.L. Burtt.

Senyumia Kiew, A. Weber & B.L. Burtt. Beitr. Biol. Pflanzen 70 (1998 [1997]) 400; Weber, Fam. & Gen. Vasc. Pl. 7 (2004) 148.

#### Type species.

*Senyumiaminutiflora* (Ridl.) Kiew, A. Weber & B.L. Burtt.

#### Revised generic description.

Short herb. **Stem** wiry, woody, to 20 cm long, 4–5 mm diameter, with a terminal rosette of many leaves. Indumentum pilose, of dense, long uniseriate hairs, intermingled with glandular hairs that make the lamina sticky to touch. **Leaves** opposite; petiole long, slender; lamina very thin, membranous, soft, pale green, broadly lanceolate to elliptic, 4–15 × 4–7.5 cm, margin toothed, teeth blunt, base not cordate, often unequal; veins 6–8 pairs. Inflorescences axillary, pair-flowered dichasial cymes, 9–17 cm long; pedicels very fine, 9–11 mm long. **Flowers** small; calyx divided to base into five narrowly lanceolate lobes, 1.5–2 mm long,; corolla white, sometimes tinged pink, tube very short, straight-sided, slightly dilating, 1.2–3 mm long, limb bilabiate, lobes in upper position densely studded with short glandular hairs internally, in non- or partially resupinate flowers, two lobes of the upper lip are strongly reflexed, in resupinate flowers, the lower three lobes held in the upper position are strongly reflexed; stamens 2, anthers large, 1.5–2 mm long, ellipsoid, cohering, yellow, exserted; nectary absent; ovary small, 2–3 mm long, ovoid; style 2–5 mm long, protruding; stigma punctiform. **Capsules** orthocarpic, 4–10 mm long, glabrous, strongly twisted, opening on the dorsal and ventral sides, valves becoming spiral after dehiscence. **Seeds** numerous, minute, 104–117 × 29–31 µm or 350 × 210 µm.

#### Distribution.

Two species, both endemic in Peninsular Malaysia.

#### Ecology.

Lithophytic, growing in cracks and crevices in light shade either on quartz derived from granite or on limestone rocks.

### Senyumia granitica

Taxon classificationPlantaeLamialesGesneriaceae

Kiew sp. nov.

urn:lsid:ipni.org:names:60478018-2

Figure 1

#### Diagnosis.

This new taxon resembles *Senyumiaminutiflora* in its wiry, woody stem; tufted leaves with an elliptic lamina with a non-cordate base; small, white flowers and orthocarpic, strongly twisted capsule. However, the new species differs from *Senyumiaminutiflora* in its shorter leaves that are less than 1.5 times longer than wide, flowers that are not resupinate or are partially resupinate, have a longer corolla tube and lobes of the lower lip that are flat and not reflexed and the much smaller seeds (Table 1).

**Table 1.** Characters that distinguish *Senyumiagranitica* from *S.minutiflora*.

Character state	* S. granitica *	* S. minutiflora *
Leaf lamina size (cm)	4–6(–10) × (3–)7–7.5	9–13 × 4–7
Ratio leaf lamina width: length	0.9-1.3	1.9-2.2
Flower position	partially or not resupinate	resupinate
Dimensions of corolla tube (mm)	2–3 × 2.5–4	1.2 × 1
Length of lower lip (mm)	4–9	2.5–3
Position of lobes of lower lip	flat (not recurved)	strongly reflexed
Length of filaments (mm)	2–3	1.5
Length of anthers (mm)	1.5–2	1.5
Capsule length (mm)	4–7.5(–10)	4–6
Seed dimensions (µm)	80–117 × 29–31	350 × 210

#### Type.

Peninsular Malaysia. Johor, Ledang District, Bukit Tukau, 10 Dec 2009, *Lau KH et al. FRI 68518* (holotype KEP!; iso.: K!, L!).

#### Description.

Perennial herb. Stem wiry, woody, ca. 4–12 cm tall, erect, 3.5–5 mm diameter. Indumentum densely woolly with long soft uniseriate, whitish hairs, dense on petiole, on lamina on upper surface and veins on lower surface, ca. 2 mm long interspersed with glandular hairs, lamina sticky to touch. **Leaves** opposite pairs spaced up to 6.5–8 cm apart; petiole fleshy, densely hairy, hairs 2–6 mm long, pale green, ca. 2 cm long in upper leaves, lengthening in lower leaves to 10.5 cm long; lamina broadly lanceolate to ovate, 4–6(–10) × (3–)7–7.5 cm, in life soft, light green above with long hairs ca. 2 mm long interspersed with glandular hairs, making the lamina sticky to touch, whitish-green beneath, base rounded, slightly to strongly unequal, apex acute, margin deeply crenate or serrate, teeth ca. 3 × 4 mm, to rounded to acute, tipped by a hair; lateral veins 6–8 pairs, deeply impressed above, prominent beneath and covered in long hairs ca. 2 mm long interspersed with glandular hairs, intercostal veins scalariform, ascending. **Inflorescences** 5–12(–17) cm long, with wispy long glandular hairs, a pair-flowered dichasium three times branched with 6–10 to many flowers; peduncle 3–9 cm, lateral branches short 1–2 cm long; pedicels erect, very fine, 8–13 mm long. **Flowers** not resupinate or partially resupinate; sepals pale green, divided almost to base, narrowly lanceolate, ca. 2 × 0.75 mm, densely glandular hairy, hairs ca. 0.5 mm long; corolla pure white, scintillating, sometimes tinged pink, ca. 7-13 mm long, outside with minute glandular hairs, tube short, not pouched, 2–3 × 2.5–4 mm, upper lip 2-lobed, 2–4 × 2.5 mm, erect, margin strongly reflexed, inner surface densely studded with short glandular hairs; lower lip glabrous, flat (not recurved) with 3 more-or-less isomorphic lobes, either lip more-or-less longer than broad, 5–9 × 6–7 mm with oblong lobes (Bukit Reban Kambing population, Fig. 1D, E) or broader than long, 4–7 × 6.5–8 mm and lobes rounded (Bukit Tukau population, Fig. 1B, C); stamens 2, filaments white, sinuous, 2–3 mm long; anthers large, pale yellow, 1.5–2 × 0.5 mm, cohering, protruding ca. 1.5 mm beyond the corolla tube; nectary absent; ovary pale green, glabrous, 2–3 × 1 mm; style white 2–5 mm, projecting beyond the corolla tube; stigma punctiform. Infructescence curling and positioned below the leaves. **Capsules** strongly twisted, 4–7.5(–10) mm long, ca. 1.5 mm diameter. **Seeds** numerous, minute, 80–117 × 29–31 µm.

#### Distribution.

Peninsular Malaysia, Johor, Ledang District, endemic on Bukit Reban Kambing and Bukit Tukau, on an isolated low range of granite hills from 200–500 m elevation, west of the southern tip of Gunung Ledang (formerly known as Mt Ophir).

#### Etymology.

From its habitat, it grows in cracks in quartz rocks derived from the granite bedrock, in contrast to *Senyumiaminutiflora* that is restricted to growing on limestone rocks.

#### Conservation status.

Critically Endangered B2a,biii. The total population probably amounts to less than 250 fertile individuals and is vulnerable because of its small and fragmented population. It is severely threatened by habitat disturbance and degradation. The forested ridge lies outside the network of Totally Protected Areas and is surrounded by oil palm plantations. The Bukit Tukau area has been logged and is currently the site of an active quarry. The area is being considered as an extension to the Gunung Ledang State Park to protect the Critically Endangered *Cycascantafolia*.

#### Ecology.

In hill dipterocarp forest on ridges at 320–505 m elevation, below the tree canopy in light shade, on low cliff faces or vertical rocky outcrops of sedimentary rocks with quartzite inter-bedded with slate that has eroded to leave horizontal cracks where this species grows. It is a rare species known only from about four small populations, each with a few to about 60 fertile plants. From the many seedlings, it is apparently regenerating freely from seed. Plants with flowers and fruits were collected in July and December but in March and October only fruiting plants with abundant fruit were found. In March 2016 after a drought, its leaves were completely wilted and dried. It is not known if these plants can recover or whether the population will regenerate from seed. The woody stem shows that they are perennial plants.

#### Notes.

There is some variation between populations. The populations on Bukit Reban Kambing have flowers that are never resupinate, are pure white and are slightly larger, the lower lip measuring 5–9 × 6–7 mm (Fig. 1D, E) compared with flowers from the Bukit Tukau population that, in some flowers, are partially resupinate (Fig. 1B), the lower lip is proportionally broader, measuring 4–7 × 6.5–8 mm and, in some individuals, the corolla is tinged pale pink (Fig. 1C). Otherwise, they are the same in calyx, corolla tube, stamen and carpel characters. It is tempting to suggest that these two forms should be given taxonomic recognition. However, very few flowers from the Bukit Tukau population were available to study variation in this population. Indeed, further investigation of the two populations may result in the recognition of two separate taxa, perhaps at an infraspecific rank.

#### Other specimens examined.

Peninsular Malaysia. Johor: Ledang District, Bukit Tukau (2°20.04'N, 102°32.3'E), 30 Oct 2009, *Lau et al. FRI 68516* (KEP!, SING!); ibidem, 27 July 2010, *Lau et al. FRI 68524* (KEP!); Ledang District, Bukit Reban Kambing (2°20.04'N 102°32.46'E), 8 March 2016, *Fakrul et al. FRI 85653* (KEP!); ibidem, 10 March 2016, *Fakrul et al. FRI 85672* (KEP!).

**Figure 2.**
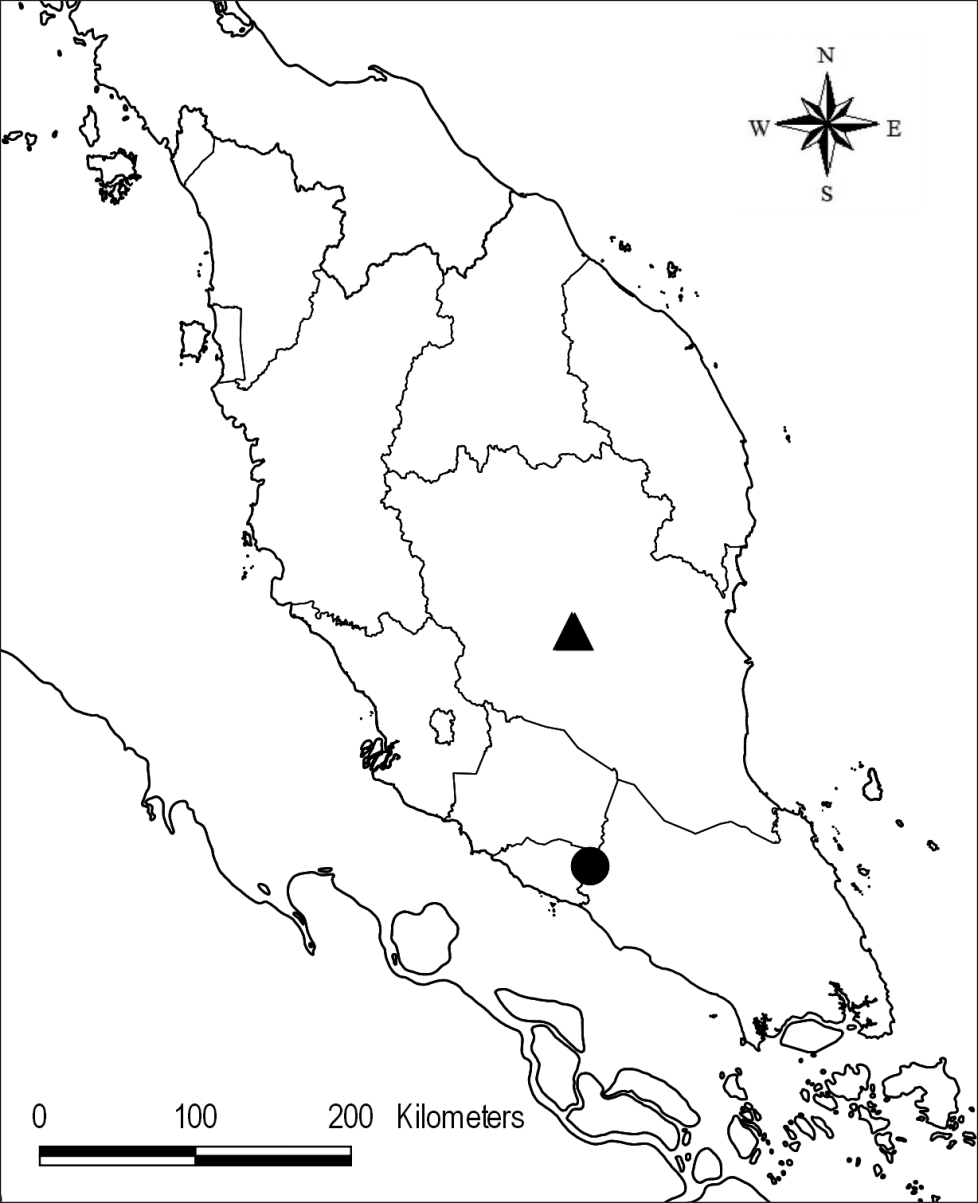
Distribution of *Senyumiagranitica* (●) and *S.minutiflora* (▲).

## Supplementary Material

XML Treatment for Senyumia

XML Treatment for Senyumia granitica
